# Investigating impact of Vascular Endothelial Growth Factor Polymorphisms in Epithelial Ovarian Cancers: A Study in the Indian Population

**DOI:** 10.1371/journal.pone.0131190

**Published:** 2015-07-09

**Authors:** Bhaskari Janardhan, Shilpa Vaderhobli, Rahul Bhagat, Premalata Chennagiri Srinivasamurthy, Pallavi Venketeshiah Reddihalli, Ramesh Gawari, Lakshmi Krishnamoorthy

**Affiliations:** 1 Department of Biochemistry, Kidwai Memorial Institute of Oncology, Bangalore, 560029, India; 2 Department of Pathology, Kidwai Memorial Institute of Oncology, Bangalore, Karnataka, India; 3 Department of Gynec-oncology, Kidwai Memorial Institute of Oncology, Bangalore, Karnataka, India; China Medical University, TAIWAN

## Abstract

Epithelial ovarian cancer is one of the increasingly incident malignancies that is notorious because of its evasiveness for early diagnosis and high mortality rates. Epithelial ovarian cancers are highly dependent on pathologic vasculature and Vascular Endothelial Growth Factor is known to be one of the most efficient angiogenic factors. Polymorphisms of the VEGF gene, in this study, were assessed for association with the malignancy and other clinico-pathological factors. 300 case samples and 320 age and mensus status matched controls were inculcated into the study. rs699947, rs833061, rs1570360, rs2010963, rs1413711 and rs3025039 were the six single nucleotide polymorphisms that were scrutinized. Genotyping was carried out by polymerase chain reaction and restriction fragment length polymorphism. rs 3025039 showed immense promise as a marker for disease aggression and recurrence and a factor for poor prognosis. rs699947 showed least association with the disease and clinico-pathologic factors studied. rs833061, rs 1570360 showed significant association with some clinico-pathological factors such as bilateral affliction of ovaries and post operative CA-125 levels. rs2010963 associated with presence of ascites in higher volumes. The SNPs under consideration showed no formidable linkage in our study samples. A haplotype analysis (excluding rs699947 and rs1413711) revealed 5 frontrunners being present in >85% of the population with TGGC and CGCC associating significantly as protective and risk factors respectively. These haplotypes showed a dose dependent additive effect of their seeming functionality. This study is unique and a first of its kind carried out in the Indian population of South-east Asia.

## Introduction

Ovarian cancer, the leading cause of death from gynaecological malignancies is the fifth most common cause of mortality in women of the Caucasian race. Survival is directly related to stage with a 5 year survival of 93% for those diagnosed with localized disease, but only 31% for those with distant disease. Sadly, two-thirds of patients present with distant disease at the time of diagnosis. In India, there has been a low incidence but a steady increase in the age-standardized prevalence rate of ovarian cancer by 3% per year in different state registries over a period of time [1].

Majority of ovarian cancers (80–90%) are epithelial ovarian tumours (EOCs). The origin and pathogenesis of EOC’s remain gallingly elusive, in part because it is rare to find well-defined precursor lesions, and in part because EOCs tend to have a complex and heterogeneous histology that defies a simple biological explanation. A lack of practical screening methods and the absence of clear symptoms in the early stages of tumour progression have made EOCs difficult to treat successfully. Paclitaxel (Taxol) and platinum-based chemotherapy (either cisplatin or carboplatin) represent standard therapy after surgical debulking. Unfortunately, even with contemporary chemotherapy, most patients with advanced disease relapse and die of the disease.

An inevitable condition that develops in tumour microenvironments is hypoxia. This is an important stimulating factor for angiogenesis, which is a propelling factor for aggressive malignancies. There are several angiogenic factors and yet Vascular Endothelial Growth Factor (VEGF-A) has been touted as the single most robust molecule in the process of angiogenesis. VEGF-A is secreted by many tumour cells invitro and is highly upregulated in most human cancers. The expression of VEGF-A correlates with intratumoral vessel density and poor prognosis in cancer patients and inhibition is known to decrease tumour vessel density and tumour growth [2, 3].

The VEGF-A gene is located on chromosome 6p12 and includes a 14-kb coding region, consists of eight exons and exhibits alternate splicing to form a family of proteins. The VEGF-A gene contains Hypoxia Responsive Elements (HREs) in its 5`and 3`UTRs. VEGF-A is upregulated under hypoxic conditions by the binding of HIF-1 (hypoxia Inducible Factor -1) to the HRE [4]. Hypoxia also mediates the stabilization of VEGF-A mRNA by binding of unidentified factors to its 3`UTR. VEGF-A is also modulated by a variety of growth factors and cytokines. Thus the gene is subject to multilevel modulation of expression under hypoxia [5]. The gene is highly polymorphic and quite a few studies have shown some of the SNPs to modulate gene expression and induce higher VEGF-A plasma levels.

Many studies have investigated the role of VEGF-A polymorphisms as a genetic determinant for susceptibility and outcome in breast, prostate, Non-Small Cell Lung Carcinoma (NSCLC), and colorectal cancer. Globally, several studies on the association of these polymorphisms with risk and treatment outcome were done but results achieved amongst different races and cancers were very variable. For example the rs3025039 ‘T’ allele imparted protection against breast cancer but conversely, increased risk of colorectal cancer. Likewise, the rs2010963 ‘C’ allele predicted higher risk of NSCLC and prostate cancer, but predicted reduced risk for colon cancer, [6]. The lack of consensus about the role of these SNPs could be because of their linkage with other obscure factors or because such SNPs modulate the expression of the gene as a whole, which has several isoforms that include both pro and anti-angiogenic isoforms making a 2-way modulation feasible by these SNPs. Perhaps, association with haplotypes will be more predictive than individual SNPs. Increased understanding of the functionality of these polymorphisms and their role in tumor biology is essential to discern the reasons for inconsistencies in the literature, which may also help in designing association studies in the future.

Polymorphisms included in this study are rs699947, rs1570360, rs833061, rs2010963, rs1413711 and rs3025039 and are commonly known as VEGFA-2578 A/C, -1154G/A, -460 C/T, +405 G/C, +674 C/T and +936 C/T, respectively. VEGFA-2578 A/C is named after its position relative to the translation start site (-1540 bp relative to the transcription start site), and is located in the promoter region. Likewise +405 G/C is also known as -634 G/C. The names for other polymorphisms are based on the positions relative to the transcription start (-460, +405) or end (+936) site, and thus these SNP are located in the promoter, 5’-untranslated (UTR) and 3’-UTR regions, respectively. +674 C/T is present in the intron 1 region of the gene. The SNPs in this paper are referred to with their respective dbSNP id numbers. These SNPs are in close proximity to the HRE region and the signalling sequence of the peptide in the gene. This puts them in the limelight for possibilities of gene expression modulation depending on the allele present.

The 6 polymorphisms of the VEGF-A gene have been included in our case-control study of epithelial ovarian cancer with the objective of investigating the role of the genetic variants as diagnostic or prognostic tools and their correlation with the disease status and clinicopathological factors. We present a first such investigation employing population from the Indian subcontinent of South-East Asia.

## Results

### Association of polymorphisms with disease

**Fig 1:**
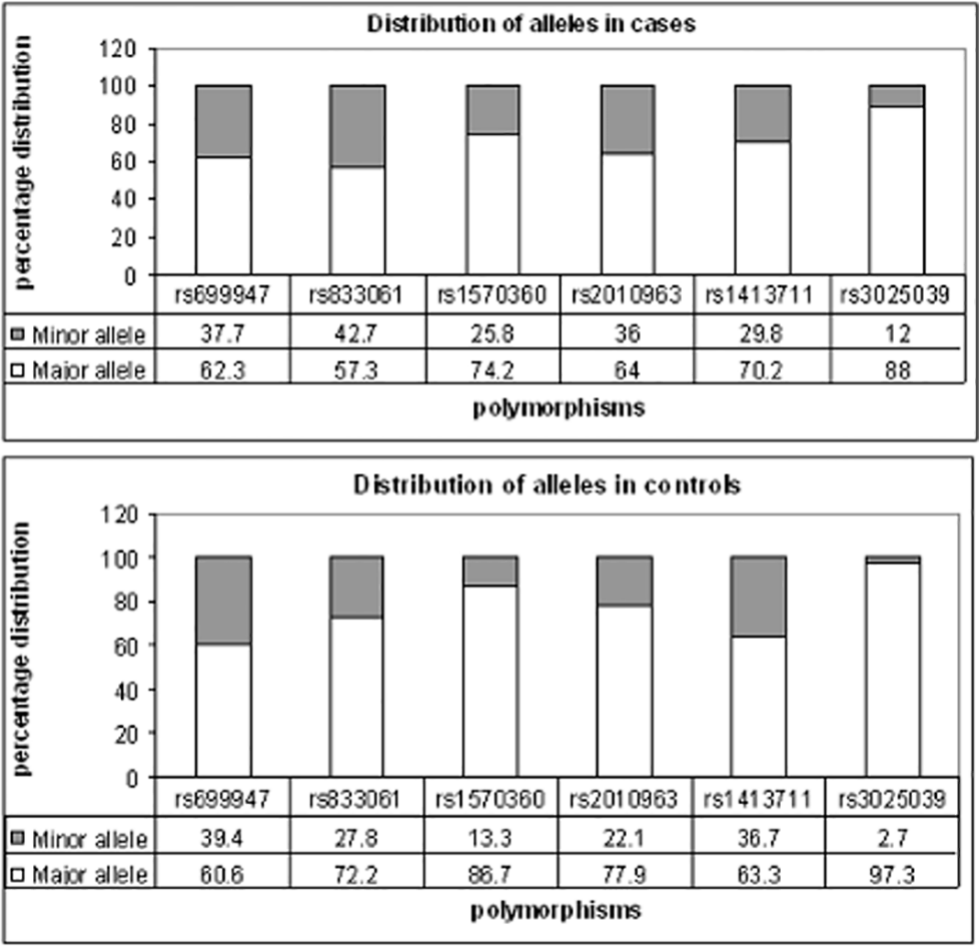
Allelic frequencies in cases and controls.

**Fig 2:**
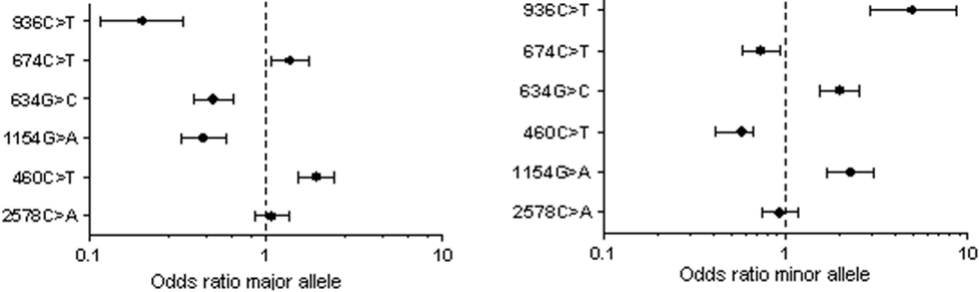
Odds ratio associated with major and minor alleles of each polymorphism.

**Fig 3:**
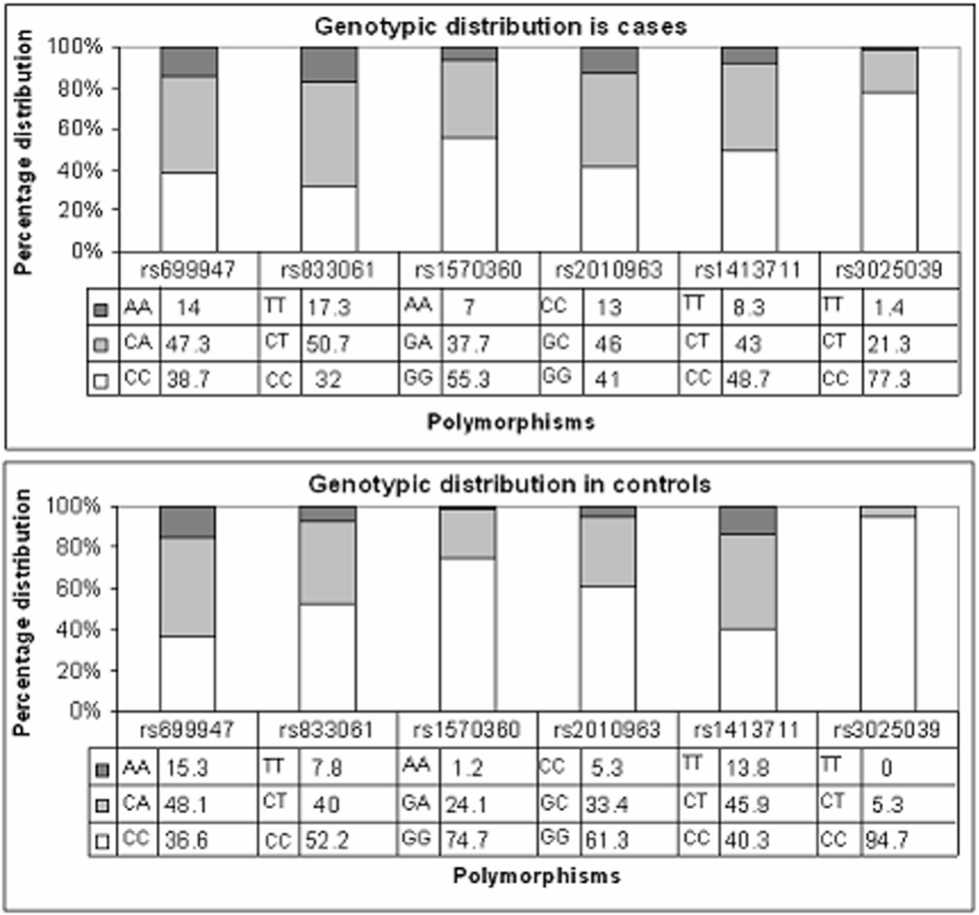
Genotypic frequencies in cases and controls.

The six polymorphisms genotyped in 300 cases and 320 controls were all found to be in Hardy-Wienberg equilibrium. The allelic distribution and association is shown in Figs 1 and 2 in terms of OR and 95% confidence interval. rs 1570360, rs 833061, rs 2010963 and rs 3025039 exhibited very strong association (p value <0.001) with disease status. rs 1413711 exhibited significant association with the disease as well (p value 0.01). rs 699947 showed no association with the disease condition in the study cohort. The genotypic distribution of the SNPs is shown in Fig 3 and an evaluation under both the dominant and recessive models for association was computed. Among the tested models, the dominant model showed higher significance of association with the disease (data shown in S1 Table).

### Association of polymorphisms with malignancy and other clinico-pathological factors

A total of eleven clinicopathological factors were included into the study like the age at diagnosis, menstrual status, FIGO stage, grade, histopathology, bilateral affliction of the ovaries, pre-operative plasma CA-125 levels, tumour type, presence or absence of ascites, presence or absence of residual disease and recurrence within a period of one year post operative surgery. (S2 Table enlists the clinicopathological factors included into the study). Post-op CA125 levels were also assessed for association with the polymorphisms to correlate with prognosis alongside recurrence, although the data for all the samples could not be retrieved. Nevertheless, an association test was done for available data subset. On an association analysis for the entire case cohort between the polymorphisms and clinico-pathological factors, SNPs rs 699947 and rs 1413711 showed no association with malignancy or disease status. All other SNPs showed significant association with malignancy with a p value of <0.001. Table 1 summarizes the results obtained for the association of the polymorphism with each of the clinicopathological factors.

**Table 1:** Association of polymorphisms with clinicopathological factors with disease status.

Characacteristics	rs699947	Rs833061	rs1570360	rs2010963	rs1413711	rs3025039
Age (mean ± SD)	0.455	0.327	0.968	0.588	0.672	0.990
Mensus status	0.247	0.655	0.441	0.531	0.367	0.226
FIGO stage	0.432	0.721	0.384	0.287	0.331	**0.012**
Grade	0.140	0.599	0.171	0.888	0.539	0.716
Histopathology	0.417	0.272	0.473	0.162	0.685	0.572
Bilateral affliction	0.392	**0.037**	0.290	0.477	0.529	0.117
Pre-op CA-125	0.856	0.719	0.776	0.614	0.661	0.903
Post-op CA-125	0.249	0.162	**0.022**	0.085	0.460	**0.001**
Ascites	0.480	0.435	0.689	0.417	**0.023**	**0.007**
Residual disease	0.656	0.245	0.578	0.494	0.171	0.218
Recurrence	0.759	0.112	0.481	0.161	0.863	**0.030**

### Linkage data and haplotype associations

**Fig 4:**
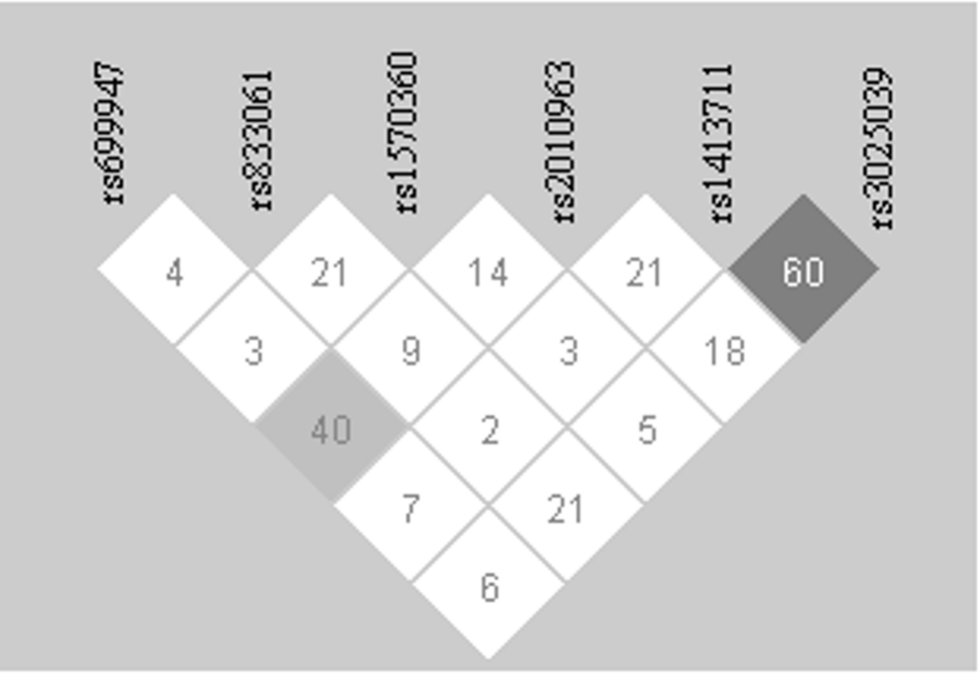
LD plot depicting the linkage between the polymorphisms included into the study.

We could not observe any significant linkage between any of the polymorphisms (Fig 4). Though rs1413711 and rs3025039 exhibit a fair extent of linkage with a D’ value of 0.605, yet it is far behind the standard cut off assumed at D’ of 0.8 or beyond. Assessing the haplotypes including all SNPs did not yield any clear fore-runner as well. When a haplotype analysis was run excluding rs699947 and rs1413711, a set of 5 haplotypes out of 16 generated through PHASE had a frequency of >5%. The haplotypes under scrutiny included the SNPs rs833061, rs1570360, rs2010963 and rs3025039. TGGC (37%), CGGC (19%), CGCC (9%), TGCC (12%) and TAGC (8%) accounted for 85% of the study population. Table 2 surmises these haplotypes, their frequency in the case–control cohorts and the tested association p-value of these haplotypes with the disease. Of these TGGC was observed at 50% of total population in controls but only 24% in cases and associated significantly as a shielding factor. CGCC was observed to be at 11% in cases but only at 6% in controls and associated significantly with disease status.

**Table 2:** Haplotype analysis and association with disease status.

Cohort	Total n	TGGC (%)	CGGC (%)	TGCC (%)	CGCC (%)	TAGC (%)
Cases	300	23.8	20	10.8	10.7	8.8
Controls	320	49.4	17.2	12.1	5.64	6.6
Total	620	36.9	18.6	11.5	8.10	7.7
P value		**<0.01**	0.367	0.614	**0.0198**	0.319

### Impact of haplotypes on disease aggression and malignancy

The haplotypes aforementioned were scrutinized for any association with disease aggression and malignancy. Haplotype TGGC associated very significantly as a protective factor (p value <0.001) in its homozygous condition. The heterozygous TGGC condition did not show any impact or association. CGCC associated significantly with malignancy (p value 0.0198). It was also observed that high malignant potentiality was observed in carriers of CGCC haplotypes in its homozygous condition but heterozygosity had no significant impact. This gives rise to the notion that the haplotypes TGGC and CGCC may have a copy-number or dose dependant effect. We observed that patients host to CGCC showed higher incidence of presence of >500ml ascites (statistical significance not achieved) and higher pre-operative plasma CA-125 levels (p value 0.017). Hosts to haplotypes CGCC and CGGC also exhibited a higher propensity to aggressive disease and malignancy. Table 3 giving an overview of the haplotype frequencies in their homozygous and heterozygous conditions and their association with disease occurrence is matriculated with Yates’ correction applied. There was no observed association between any of the haplotypes with recurrence of disease within a period of one year nor was it associated with residual disease status in the current study.

**Table 3:** Evaluation of haplotype copy number effect on disease status.

Haplotypes	Zygosity	Cases (n = 300)	Controls (n = 320)	P value	Corrected P Value
TGGC	Homozygous	14	89	**<0.001**	**<0.001**
	Heterozygous	143	149	0.783	0.846
CGGC	Homozygous	18	12	0.192	0.264
	Heterozygous	112	95	**0.044**	0.053
TGCC	Homozygous	2	6	0.183	0.329
	Heterozygous	29	43	0.143	0.181
CGCC	Homozygous	2	1	0.525	0.955
	Heterozygous	63	41	**0.006**	**0.009**
TAGC	Homozygous	0	0	NA	NA
	Heterozygous	43	42	0.662	0.749

## Discussion

The need for a vascular support system is essential for growth, be it tumour cells or normal cells. Tumour angiogenesis is a primary event that fulfills this need without which tumour cells cannot grow beyond 2mm and stay dormant [7]. An intricate balance between pro and anti- angiogenic factors is necessary for healthy angiogenesis. EOCs, being angiogenesis-dependent, require an angiogenic switch that tilts the balance favoring angiogenesis [8]. In advanced ovarian cancer, VEGF-A induced pathologic angiogenesis leads to malignant ascites production and eventual disease progression and treatment failure. A plethora of SNPs in the VEGF-A gene have been discovered that have been associated with modulating levels of VEGF-A protein expression and susceptibility to various carcinomas [9–13]. Studies interrogating the effect of polymorphisms and testing their validity as prognostic markers or risk factors are far and few and most of them in Caucasian races. Studies employing the Asian subset are sparse.

Amongst the few studies in ovarian cancers, rs3025039 was observed to be associated as a risk factor in one study [14] and was associated with better prognosis in another study [15]. Our study implicated the SNP with poor prognosis and risk factor as in the former study. rs2010963 associated strongly with better prognosis but weak association in case of rs803361 [16]. The study also showed that the zygosity held independent prognostic significance. A study including rs2010963, rs1570360 and rs699947 indicated no significant association of the polymorphisms with any clinico-pathological parameters but associated with significant alteration of VEGF-A expression but no association with progression free survival or prognosis and suggested significant linkage disequilibrium amongst these polymorphisms [17]. The haplotype analysis in the study showed no significant association with clinico-pathological parameters although one of their haplotypes AGCGC (rs699947, rs1570360, rs833061, rs2010963 and rs3025039) showed significant association with progression free survival.

The SNPs in our study though exhibited strong association with the disease status, did not show any significant linkage between themselves as reported in several other studies [18–22]. Several of the SNPs included showed significant association with certain clinico-pathological parameters. rs3025039 showed significant associations with malignant potentiality, FIGO stages, bilateral affliction, post-op CA125 plasma levels, ascites and recurrence indicating it to be promising marker for poor prognosis. rs1413711 and rs2010963 associated significantly with presence of ascites but significance was not seen with increased levels of ascites. rs833061 associated with bilateral affliction of ovaries and even more strongly within the malignant subset of case samples. rs699947 showed no association to disease status or other clinico-pathological parameters.

The ambiguity in polymorphism data across populations could partly be explicated in terms of the positions of these well reported polymorphisms and to the isoforms of VEGF-A that are known to be anti-angiogenic. As much as an angiogenic switch occurs in favour of angiogenesis in the event of tumorigenesis, there is also a self-regulatory mechanism with respect to the VEGF-A gene that maintains a fine balance between the pro- and anti- angiogenic isoforms of the VEGF-A gene.

Exon 8 of the VEGF-A gene has a unique capability of producing both pro and anti angiogenic isoforms of VEGF-A. While exon 8a confers pro-angiogenecity, exon 8b confers anti-angiogenecity. Alternative splicing that produces mRNA with 8b codons produce an entire set of isoforms of VEGF-A that are completely contradicting the effects of the mRNA of 8a subsets. Perrin et al have reported that expression of VEGF-A_xxxb_ isoforms contribute to competitive inhibition by binding to the receptors but not stimulating tyrosine phosphorylation essential for downstream angiogenic activity as is caused by VEGF-A_xxxa_ isoforms. The VEGF-A_xxxb_ isoforms are hence endogenous anti-angiogenic agents formed by alternative splicing and are said to be influenced by other growth factor conditions and splice factor availabilities. The VEGF-A_165b_ isoforms have been reported to be down-regulated in various cancers such as renal cell carcinoma, prostate and colon carcinoma [23–25].

This particular aspect of VEGF-A splicing and alternate isoform production is definitely worth a detailed study since explorative studies on SNPs worldwide has not yielded a consensus data that is implicative. Part reason to this is the varying condition in-vivo in cancer patients. EOCs are even more complicated since a recent report showed them to be of more mixed origins rather than just surface epithelium [26]. This heterogenous invivo mileu makes the modulation of VEGF-A gene expression highly intricate.

The SNPs rs 699947, rs 833061 and rs 1550360 at the promoter region are common for both the VEGF-A_xxxa_ and VEGF-A_xxxb_ isoforms and hence an association of these SNPs with the disease could mean either ways. One, it could concur that the pro-angiogenic isoforms are increasingly expressed or it could also mean that the anti-angiogenic isoforms are being suppressed. SNP rs 2010963 is within the 5’ UTR region. The VEGF-A gene is singular in the fact that it has a 1038bp 5’UTR region that has 2 IRES (Internal ribosomal entry sites) elements (IRES-A and IRES-B) that is differentially put into use depending on the intracellular conditions of normoxia and hypoxia [27]. Hence the effect of rs 2010963 could turn indistinct considering the novel fact that a long form of VEGF-A mRNA (with an added 180 amino acid sequence) is produced that modulates the further expression of VEGF-A mRNAs. rs 1413711 is, included in this study, is not well studied as the other polymorphisms but is yet worth a watch, being in the intron 1 region. In our study, it did not exert any significant association with the clinicopathological factors but did associate with considerable significance (p = 0.01) with the disease status. SNP rs 3025039 is well documented, being in the 3’ UTR region of the VEGF-A gene. It is known to influence the secreted levels of VEGF-A protein and has been shown to have significant association in most of the case-cohort studies. This SNP, in our study too exhibited very strong association with disease status and poorer prognosis.

This investigative study is a first in its attempt in this population and in summary shows rs3025039 to be associated as a highly promising marker for prognosis while other polymorphisms in the study were associated with a few clinico-pathological parameters and were inconclusive about their prognostic capabilities. The polymorphisms did show a strong association with malignancy and hence can be implicated as risk factors. This summarily is the only paper that addresses the effect of VEGF-A polymorphisms in epithelial ovarian cancer in the South East Asian population, specifically pan-Indian.

## Materials and Methods

### Sample collection

A total of 300 patients diagnosed with primary epithelial ovarian tumours at the Kidwai Memorial Institute of Oncology, Bangalore, India, were included into the study.

The initial institutional diagnosis of epithelial ovarian cancer was confirmed by review of pathologic slides by senior pathologists. Of the 300 cases, 202 (67.3%) were malignant cases, 31 (10.3%) were borderline or Low Malignant Potential (LMP) tumours and 67 (22.4%) were benign tumours. Of these, number of pre-mensus samples stood at 88 (29.3%) and post-mensus samples at 212 (70.7%). 320 healthy control subjects, matched for age and menopausal status, were recruited for the study. The average age of case cohort was 51 ± 12 years and 48 ± 17 years for controls. 30.9% (99/320) of controls were pre-menopausal and 69.1% (221/320) were post-menopausal. 65.8% cases were high stage and high grade, and 63.8% cases were serous epithelial ovarian cancers. 64.9% cases had bilateral affliction and 86.6% cases showed high CA125 levels. Ascites was observed in 80% cases. S2 Table in the gives an in-detail view of the demography of the cases taken into the study. 5ml of blood was collected from all subjects, prior to treatment, in heparin vacutainers and processed immediately there after. The cell pellet was stored in -20°C for DNA isolation and genotyping.

All tumors were graded according to WHO criteria and staged according to the Federation of Gynecology and Obstetrics (FIGO) classification. Information on cancer diagnosis, disease stage (FIGO), histological grade and subtype, pre operative CA-125 levels, residual disease and recurrence were abstracted from medical records.

Study approval was given by the Institutional Review Board and the Medical Ethics Committee and written informed consent was obtained from all participants.

### DNA isolation

Genomic DNA was extracted from peripheral blood sample using QIAamp® DNA extraction kits (QIAGEN) as per manufacturer’s protocol. DNA concentration was measured by nanodrop from thermo scientific.

### Genotyping

Polymerase Chain Reaction—Restriction Fragment Length Polymorphism (PCR-RFLP) was used to determine the 6 VEGF-A polymorphisms using previously published protocols with minor modifications.

The PCR was carried out in a 30μl reaction mix with 50–100ng template DNA, 1U of Taq Polymerase enzyme (Bangalore Genei) and 1X buffer provided along with enzyme. 0.3μM of respective primers (Sigma), 0.5mM dNTP mix and 1.5 to 2 mM MgCl_2_ constituted the PCR mix. The PCR was carried out in an Eppendorf PCR thermal cycler with an initial denaturation at 95°C for 5 mins followed by 35–40 cycles of denaturation at 95°C for 40 seconds, annealing for 40 seconds, extension at 72°C for 20–40 seconds and a final extension for five minutes at 72°C. The PCR primers and respective annealing temperatures used for each SNP in listed in Table 4.

**Table 4:** PCR primers and annealing temperatures.

SNP	Forward Primer	Reverse Primer	Ann temp
rs 699947	GGGCCTTAGGACACCATACC	TGCCCCAGGGAACAAAGT	57°C
rs 833061	TGTGCGTGTGGGGTTGAGCG	TACGTGCGGACAGGGCCTGA	60°C
rs 1570360	CTTGGTGGGGGTCGAGCT	GGACAGGCGAGCCTCAGC	58°C
rs 2010963	TTGCTTGCCATTCCCCACTTGA	GGGCGGTGTCTGTCTGTCTG	65°C
rs 1413711	CGCAAGTTCCTCAGACCC	ACCCATTCCCATGACACC	61°C
rs 3025039	AGGAAGAGGGACTCTGCGCAGAGC	TAAATGTATGTATGTGGGTGGGTGTGTCTACAG	64°C

The obtained DNA amplicons were digested overnight with the restriction enzyme. The restriction enzymes were procured from New England Biolabs and restriction temperature conditions followed manufacturer’s protocol. The enzymes used were BglII (rs699947), BstUI (rs833061), MnlI (rs1570360), BsmFI (rs 2010963), BaeGI (rs 1413711) and NlaIII (rs3025039). BstUI was used at 2U concentration while the rest of the enzymes were used at a 1U concentration in a reaction mix volume of 25μl. The digested amplicons were separated in an ethidium bromide gel ranging from 1.5 to 3% agarose depending on the amplicon size. The band sizes were ascertained with the use of a 50bp and 100bp ladder.

### Linkage, haplotype and statistical analysis

The linkage and haplotype analysis was carried using haploview 4.2 and phase 2.1.1 version. The χ^2^ test was applied to determine whether the genotype distributions were within the Hardy–Wienberg equilibrium. Chi square and logistic regression analyses were applied to examine association between the carriage of markers and disease status and other clinicopathological factors. All statistical analysis were done using SPSS version 21.

## Supporting Information

S1 Table An analysis of the polymorphisms under the dominant and recessive models. (DOC)

S2 Table Demography and clinicopathological aspects of cases (total 300). (DOC)
