# Supplementary material for: Investigating impact of Vascular Endothelial Growth Factor Polymorphisms in Epithelial Ovarian Cancers: A Study in the Indian Population
Source: PLoS One. 2015 Jul 9;10(7):e0131190. doi: 10.1371/journal.pone.0131190 (PMC4497663; doi:10.1371/journal.pone.0131190)
Supplement: S1 Table — (DOC) [file pone.0131190.s001.doc]

**Supplementary section**

**Table S1**: An analysis of the polymorphisms under the dominant and recessive models.

| SNP name | dbSNP id | Genotype | OR(95%CI) | P value |
| --- | --- | --- | --- | --- |
| 2578C>A | rs 699947 | CC Vs CA + AA | 1.094(0.790,1.514) | 0.589 |
|  |  | AA Vs CA + CC | 0.645(0.576,1.406) | 0.645 |
| 460C>T | rs 833061 | CC Vs CT + TT | 0.431(0.311,0.598) | **<0.001** |
|  |  | TT Vs CT + CC | 2.474(1.492,4.104) | **<0.001** |
| 1154G>A | rs 1570360 | GG Vs GA + AA | 0.42(0.299,0.590) | **<0.001** |
|  |  | AA Vs GA + GG | 5.946(2.017,17.53) | **=0.001** |
| 634G>C | rs 2010963 | GG Vs GC + CC | 0.440(0.319, 0.606) | **<0.001** |
|  |  | CC Vs GC +GG | 2.663(1.472,4.82) | **=0.001** |
| 674C>T | rs 1413711 | CC Vs CT + TT | 1.404(1.021, 1.929) | **0.037** |
|  |  | TT Vs CT + CC | 0.570(0.340,0.958) | **0.034** |
| 936C>T | rs 3025039 | CC Vs CT + TT | 0.191(0.110, 0.335) | **<0.001** |
|  |  | TT Vs CT + CC | --- | 0.054 |
