# Supplementary material for: Investigating impact of Vascular Endothelial Growth Factor Polymorphisms in Epithelial Ovarian Cancers: A Study in the Indian Population
Source: PLoS One. 2015 Jul 9;10(7):e0131190. doi: 10.1371/journal.pone.0131190 (PMC4497663; doi:10.1371/journal.pone.0131190)
Supplement: S2 Table — (DOC) [file pone.0131190.s002.doc]

**Supplementary section**

**Table S2**: Demography and clinicopathological aspects of cases (total 300)

| Characacteristics | Malignant  n(%) | Borderline (LMP)  n(%) | Benign  n(%) | Total  n(%) | P value |
| --- | --- | --- | --- | --- | --- |
| Number (n) | 202 (67.3) | 31 (10.3) | 67 (22.4) | 300 (100) |  |
| Age (mean ± SD) | 51 ± 11 | 46 ± 16 | 50 ± 14 | 51 ± 12 | **0.0015** |
| Mensus status |  |  |  |  | **<0.001** |
| Pre mensus | 51 (25.2) | 15 (48.4) | 22 (32.8) | 88 (29.3) |  |
| Post mensus | 151 (74.8) | 16 (51.6) | 45 (67.2) | 212 (70.7) |  |
| FIGO stage |  |  |  |  | **<0.001** |
| I and II | 69 (34.2) | 31 (100) | NA | 100 (33.3) |  |
| III and IV | 133 (65.8) | 0 (0) | NA | 133 (44.3) |  |
| Grade |  |  |  |  | **<0.001** |
| I and II | 67 (33.2) | 29 (93.5) | NA | 96 (32) |  |
| III and UD | 135 (66.8) | 2 (6.5) | NA | 137 (45.6) |  |
| Histopathology |  |  |  |  | **<0.001** |
| Serous | 128 (63.4) | 10 (32.3) | 38 (56.7) | 176 (58.7) |  |
| Mucinous | 17 (8.4) | 19 (61.3) | 28 (41.8) | 64 (21.3) |  |
| Endometrioid | 7 (3.5) | 2 (6.4) | 1 (1.5) | 10 (3.3) |  |
| Clear cell | 11 (5.4) | 0 (0) | 0(0) | 11 (3.7) |  |
| Poorly differentiated | 39 (19.3) | 0 (0) | 0(0) | 39 (13) |  |
| Bilateral affliction | 131 (64.9) | 8 (25.8) | 9 (13.4) | 148 (49.3) |  |
| Pre Op CA125 |  |  |  |  | **<0.001** |
| 0-35 | 8 (3.9) | 10 (32.3) | 33 (49.2) | 51 (17) |  |
| 35-110 | 11 (5.5) | 9 (29) | 20 (29.9) | 40 (133) |  |
| 110-100 | 98 (48.5) | 11 (35.5) | 13 (19.4) | 122 (40.7) |  |
| >1000 | 85 (42.1) | 1 (3.2) | 1 (1.5) | 87 (29) |  |
| Ascites |  |  |  |  | **<0.001** |
| Nil | 40 (19.8) | 13 (41.9) | 52 (77.6) | 105 (35) |  |
| <500ml | 68 (33.7) | 16 (51.6) | 15 (22.4) | 99 (33) |  |
| >500ml | 94 (46.5) | 2 (6.5) | 0(0) | 96 (32) |  |
| Residual disease |  |  |  |  | ND |
| Nil | 75 (37.1) | 25 (80.6) | 64 (95.5) | 164 (54.7) |  |
| <1 | 54 (26.7) | 2 (6.5) | 2 (3) | 58 (19.3) |  |
| >1 | 32 (15.9) | 0(0) | 0(0) | 32 (10.7) |  |
| Not known | 41 (20.3) | 4 (12.9) | 1 (1.5) | 46 (15.3) |  |
| Recurrence |  |  |  |  | ND |
| Yes | 41 (20.3) | 1 (3.2) | 1 (1.5) | 43 (14.3) |  |
| No | 69 (34.2) | 14 (45.2) | 1 (1.5) | 84 (28) |  |
| Not known | 92 (45.5) | 16 (51.6) | 65 (97) | 173 (57.7) |  |

NA – Not applicable, ND – Not Determined
